# *Paeonia lactiflora* Enhances the Adhesion of Trophoblast to the Endometrium via Induction of Leukemia Inhibitory Factor Expression

**DOI:** 10.1371/journal.pone.0148232

**Published:** 2016-02-03

**Authors:** Hee-Jung Choi, Tae-Wook Chung, Mi-Ju Park, Kyu Sup Lee, Youngjin Yoon, Hyung Sik Kim, Jun Hee Lee, Sang-Mo Kwon, Syng-Ook Lee, Keuk-Jun Kim, Jin-Ho Baek, Ki-Tae Ha

**Affiliations:** 1 Department of Korean Medical Science, School of Korean Medicine and Korean Medicine Research Center for Healthy Aging, Pusan National University, Yangsan, Gyeongsangnam-do, Republic of Korea; 2 Department of Obstetrics & Gynecology, Pusan National University Hospital, Pusan, Republic of Korea; 3 Department of Korean Obstetrics & Gynecology, School of Korean Medicine, Pusan National University, Pusan, Republic of Korea; 4 Laboratory of Molecular Toxicology, School of Pharmacy, SungKyunKwan University, Suwon, Gyeonggi-do, Republic of Korea; 5 Laboratory for Vascular Medicine and Stem Cell Biology, Department of Physiology, Medical Research Institute, School of Medicine, Pusan National University, Yangsan, Gyeongsangnam-do, Republic of Korea; 6 Department of Food Science and Technology, Keimyung University, Daegu, Republic of Korea; 7 Department of Clinical Pathology, TaeKyeung University, Gyeongsan, Republic of Korea; 8 Daechubatbaek Korean Medical Clinic, Gyeongju, Gyeongsangbuk-do, Republic of Korea; National Institute for Research in Reproductive Health, INDIA

## Abstract

In the present study, we investigated the role of *Paeonia lactiflora Pall*. extract on embryo implantation *in vitro* and *in vivo*. A polysaccharides depleted-water extract of *P*. *lactiflora* (PL-PP) increased LIF expression in human endometrial Ishikawa cells at non-cytotoxic doses. PL-PP significantly increased the adhesion of the human trophectoderm-derived JAr spheroids to endometrial Ishikawa cells. PL-PP-induced LIF expression was decreased in the presence of a p38 kinase inhibitor SB203580 and an MEK/ERK inhibitor U0126. Furthermore, endometrial LIF knockdown by shRNA reduced the expression of integrins β3 and β5 and adhesion of JAr spheroids to Ishikawa cells. *In vivo* administration of PL-PP restored the implantation of mouse blastocysts in a mifepristone-induced implantation failure mice model. Our results demonstrate that PL-PP increases LIF expression via the p38 and MEK/ERK pathways and favors trophoblast adhesion to endometrial cells.

## Introduction

Implantation is a complex biological process, requiring communication between the appropriately developed trophoblast and the receptive endometrium [1]. This process is established and maintained by diverse biological factors, including cytokines, growth factors, and receptors [2]. Embryo implantation is adversely affected by abnormal expression of the genes related to the establishment of uterine receptivity [3]. Among these factors, leukemia inhibitory factor (LIF) plays a key role in determining the outcome of implantation [4–6]. Genetic mutations and aberrant expression of LIF are known to contribute to implantation failure in mice and humans [7,8]. In addition, inhibition of LIF activity by addition of an anti-LIF antibody or LIF antagonist effectively prevents embryo implantation in mice, monkeys, and humans [9–12].

In recent years significant advances have been made in assisted reproductive technologies (ART). However, pregnancy rates remain low [13]. High doses of exogenous gonadotropins used for ovarian stimulation during *in vitro* fertilization (IVF) procedures are known to impair endometrial receptivity [14]. Traditional herbal remedies and acupuncture, therefore are being proposed as alternative treatments for improving endometrial receptivity [15–17]. Several groups have reported that LIF is an important molecular target of herbal remedies and acupuncture [18–20].

The roots of *P*. *lactiflora Pall*., belonging to the Paeoniaceae family, have been used as a therapeutic drug for treating fever, rheumatoid arthritis, systemic lupus erythematosus, hepatitis, and gynecological disorders in Korea, Japan, and China [21,22]. Especially, *P*. *lactiflora* has been used for several years to treat various gynecological problems such as dysmenorrhea, cramps and spasms during pregnancy, and infertility [23,24]. Different components extracted from the roots of *P*. *lactiflora* are reported to have anti-inflammatory, immunomodulatory, anti-allergic, anti-arthritic, and hepatoprotective activities [21,25–27]. However, the biological effects of *P*. *lactiflora* extract on endometrial receptivity have not been explored.

Therefore, in this study, we evaluated the effects of *P*. *lactiflora* extract on the LIF expression in endometrial Ishikawa cells and adhesion of trophoblastic JAr spheroids to Ishikawa cells. In addition, the effect of *P*. *lactiflora* extract on embryo implantation was examined in an implantation- failure mouse model.

## Materials and Methods

### Materials

Antibodies against LIF, extracellular signal-regulated kinase (ERK), p-ERK, p38, signal transducer and activator of transcription 3 (STAT3), p-STAT3, and β-actin were purchased from Santa Cruz Biotechnology (Santa Cruz, CA, USA). Anti-p-p38 was obtained from Cell Signaling Technology (Danvers, MA, USA). Inhibitors of specific signaling pathways, including LY294002 (inhibitor for Phosphoinositide 3-Kinase; PI3K), U0126 (inhibitor for Mitogen-Extracellular signal-regulated Kinase; MEK/ERK), SB203580 (inhibitor for p38), and SP600125 (inhibitor for c-Jun N-terminal Kinase; JNK), were obtained from Merck Millipore (Billerica, MA, USA). An antagonist of the progesterone receptor, mifepristone (RU486), was purchased from Sigma-Aldrich (St. Louis, MO, USA).

### Extract preparation

The roots of *P*. *lactiflora* were purchased from Omniherb Co. (Daegu, Korea). The herb was authenticated by a botanist at Omniherb Co. A voucher specimen (DC-H21) in the form of a frozen rhizome was deposited in the Korean Medicine Research Center for Healthy Aging, Pusan National University (Yangsan, Korea). The extraction method is described in S1 Fig. PL-WE and PL-PP were freshly dissolved in distilled water before performing subsequent experiments.

### Fingerprinting high-performance liquid chromatography (HPLC) analysis

HPLC analysis was performed using a Shimadzu HPLC system (Shimadzu Co., Kyoto, Japan), consisting of a solvent delivery unit, an on-line degasser, a column oven, an autosampler, and an SPD-20A UV detector. For data analysis, LC solution software (Version 1.25) was used. The analytical column used was an ACE C18 column (4.6 × 250 mm i.d., 5 μm pore size). The mobile phases were solvent A (acetonitrile) and solvent B (0.1% phosphoric acid). The gradient flow was as follows: (A)/(B) = 10–15/90–85 (0–5 min) → (A)/(B) = 15–22/85–78 (5–25 min) → (A)/(B) = 22–70/78–30 (25–45 min) → (A)/(B) = 70–80/30–20 (45–46 min) → (A)/(B) = 80/20 (46–50 min). The column temperature was maintained at 25°C. The analysis was carried out at a flow rate of 1 mL/min with UV detection at 254 nm. The column injection volume was 20 μL. The standard solution and PL-PP were prepared by dissolution in dimethyl sulfoxide (DMSO; 1 mM and 50 mg/mL, respectively). The solutions were filtered through a 0.45-μm membrane filter (Millipore) and applied to HPLC.

### Cell culture

Human endometrial Ishikawa cell line, derived from a human adenocarcinoma, was kindly provided by Dr. Jacques Simard (CHUL Research Center, Quebec, Canada). The cells were maintained as monolayers at 37°C in an atmosphere containing 5% CO_2_/air in Dulbecco’s Modified Eagle Medium (DMEM; Welgene, Daegu, Korea) containing 10% heat-inactivated fetal bovine serum (FBS, Sigma-Aldrich) and 1% penicillin/streptomycin (Gibco, Rockville, MD, USA). The human choriocarcinoma JAr cells were obtained from the Korean Cell Line Bank (Seoul, Korea) and cultured in RPMI1640 (Welgene) containing 10% heat-inactivated FBS and 1% penicillin/streptomycin. AN3CA cell line was purchased from American Type Culture Collection (ATCC, Manassas, VA, USA).

### Cell viability Assay

The cytotoxic effects of PL-WE and PL-PP were examined using the methylthiazolyldiphenyl-tetrazolium bromide assay (MTT, Sigma-Aldrich). In brief, Ishikawa cells were cultured in 24-well plates with PL-WE and PL-PP (indicated concentrations) for 24 h. After removal of the culture medium, 300 μL of 1× MTT solution (0.5 mg/mL) was added to each well. After incubation for 4 h at 37°C in a CO_2_ incubator, the supernatants were removed and formazan crystals formed in viable cells were dissolved in 300 μL of ethanol:DMSO (v/v, 1:1). To detect the cytotoxicity, the absorbance in each well was measured at 540 nm with a microplate reader. The percentage of living cells in treated cultures was calculated relative to that in untreated cultures.

### Reverse transcription-polymerase chain reaction (RT-PCR)

Total RNA was isolated from Ishikawa cells using RiboEx^™^ (GeneAll, Seoul, Korea). Equal amounts of total RNA (1 μg) from each sample were then subjected to reverse transcription with oligo-dT primers by using M-MLV reverse transcriptase (Enzynomics, Daejeon, Korea). The cDNA was amplified by PCR using DiaStar^™^ Taq DNA Polymerase (Solgent Co., Daejeon, Korea). The PCR conditions, amplified size of each target gene, and primers used in this study are shown in Table 1.

**Table 1:** PCR conditions and amplified size for each target gene, and the primers used in this study.

Gene	Primer Sequences	PCR conditions	Size (bp)
*LIF*	Forward: 5'-GGCCCGGACACCCATAGACG-3'	60°C	455
	Reverse: 5'-CCACGCGCCATCCAGGTAAA-3'	30 cycles	
*ITGAV*	Forward: 5'-ATGCTCCATGTAGATCACAAGAT-3'	60°C	339
	Reverse: 5'-TTCCCAAAGTCCTTGCTGCT-3'	32 cycles	
*ITGB1*	Forward: 5'-GTCGTGTGTGTGAGTGCAAC-3'	60°C	318
	Reverse: 5'-GCTGGGGTAATTTGTCCCGA-3'	30 cycles	
*ITGB3*	Forward: 5'-CTGCCGTGACGAGATTGAGT-3'	60°C	383
	Reverse: 5'-TGCCCCGGTACGTGATATTG-3’	32 cycles	
*ITGB4*	Forward: 5'-GAGCTCACCAACCTGTACCC-3'	60°C	262
	Reverse: 5'-GCCCAATAGGTCGGTTGTCA-3'	30 cycles	
*ITGB5*	Forward: 5'-ACCTGGAACAACGGTGGAGA-3'	60°C	217
	Reverse: 5'-AAAAGATGCCGTGTCCCCAA-3'	32 cycles	
*CD44*	Forward: 5'-AGGGATCCTCCAGCTCCTTT-3'	62°C	466
	Reverse: 5'-AAAGGCATTGGGCAGGTCTGTGACT-3'	28 cycles	
*ICAM-1*	Forward: 5'-CAGTGACCATCTACAGCTTTCCGG-3'	60°C	556
	Reverse:5'-GCTGCTACCACAGTGATGATGACAA-3'	32 cycles	
*L-selectin*	Forward: 5'-AAACCCATGAACTGGCAAAG-3'	60°C	250
	Reverse:5'-CGCAGTCCTCCTTGTTCTTC-3'	35 cycles	
*E-cadherin*	Forward: 5'-TACAATGCCGCCATCGCTTA-3'	60°C	470
	Reverse:5'-AGCTGTGAGGATGCCAGTT-3'	32 cycles	
*β-actin*	Forward: 5'-CAAGAGATGGCCACGGCTGCT-3'	60°C	275
	Reverse: 5'-TCCTTCTGCATCCTGTCGGCA-3'	25 cycles	

### Western blot analysis

The total protein was isolated from Ishikawa cells exposed to PL-WE or PL-PP (indicated concentrations). Protein (20 μg) from each sample was boiled with 6× sodium dodecyl sulfate (SDS) sample loading buffer (0.35M Tris-HCl [pH 6.8], 10% SDS, 30% glycerol, 9.3% dithiothreitol [DTT], and 12% bromophenol blue), and then separated using 10% sodium dodecyl sulfate polyacrylamide gel electrophoresis (SDS-PAGE) gel. The size-fractioned protein sample was electro-transferred to nitrocellulose membranes. The membranes were blocked using 5% blocking solution and incubated with anti-LIF, p-Erk, Erk, p-p38, p38, and anti-β-actin antibodies. After reaction with the appropriate secondary antibodies linked to horseradish peroxidase, signals were visualized using the ECL chemiluminescence system (GE Healthcare, Uppsala, Sweden).

### *LIF* knockdown by shRNA

To knock down endogenous hLIF, lentiviral shRNA constructs were obtained from Open Biosystems (Thermo Scientific, Waltham, MA, USA). 293T cells (1.5 × 10^6^) were subcultured in a 10 cm dish. Twenty-four hours after cell seeding, the lentiviral vector encoding hLIF shRNA (3 μg), packaging vector pCMV-VSVG (0.3 μg), and the envelop plasmid pHRCMV-8.2 ΔR (3 μg) were transfected in 293T cells using Lipofectamine 2000 (Invitrogen, Carlsbad, CA, USA). Forty-eight hours after transfection, the supernatant harboring lentiviruses was collected and filtered using a sterilized 0.45 μm syringe filter. LIF shRNA lentiviral supernatant with polybrene (5 μg/mL) was added to the Ishikawa cells. To select cells with stable integration of LIF shRNA, the viral infected Ishikawa cells were treated with 3 μg/mL puromycin for 1 week. The knockdown efficiency of the LIF shRNA was verified by western blot analysis. The best hLIF shRNA #5, among 5 hLIF shRNA clones (#3–#7), was used for subsequent experiments. The sequence of LIF shRNA #5 used for knocking down *LIF* was 5’-TTACCCGAGGTGTCAGGGCCG-3’.

### JAr spheroid adhesion assay

Ishikawa cells (3 × 10^5^ cells) were seeded into 24 well plates and cultured for 24 h. The medium was replaced and the cells were incubated in serum-free -DMEM containing PL-PP for 48 h. To make JAr spheroids, we used the GravityPLUS™ Kit (InSphero Co.) according to the manufacturer’s manual. Briefly, after JAr cells were trypsinized and counted, the suspended JAr cells (1 × 10^3^ /40 μl RPMI medium) were gently delivered into each well of the GravityPLUS™ plate and incubated in a humidified 5% CO_2_ incubator at 37°C for 3 days. JAr spheroids (20/well) were gently delivered onto a confluent monolayer of Ishikawa cells grown in a 24 well culture plate. After incubation for 2 h at 37°C, the cells were washed with 10% FBS/RPMI medium three times to remove non-binding JAr spheroids. The attached JAr spheroids were visualized using a camera and counted.

### Animals

Male and female C57BL/6 mice (7–8 weeks old, weight 20–22 g), inbred in a specific pathogen-free (SPF) facility, were purchased from Orient Bio, Co. (Seongnam, Korea). They were bred separately and had free access to water and a standard diet with a 12 h light / 12 h dark cycle. All experimental procedures were examined and approved by the Animal Research Ethics Committee at the Pusan University of Korea.

### Animal models and treatment

All 33 female mice were randomly divided into 3 groups, including a control group, RU486 group, and RU486 with PL-PP group. To improve implantation, the female mice were treated with PL-PP every day for 17 days. Female mice belonging to the RU486 with PL-PP group received PL-PP orally (1.06 mg/20 g) every day, while the mice belonging to the control and RU486 groups received the same amount of PBS. After seven days of daily treatment with PL-PP, all females were caged with males (ratio 2:1) at 6 pm and the day when vaginal plugs were detected was designated as day 1 of pregnancy. The embryo implantation failure model was developed according to previous reports with some modifications [20,28]. Briefly, the mice belonging to the RU486 group and RU486 with PL-PP group were injected subcutaneously with 0.08 mg/0.1 mL of RU486 solution, while the mice belonging to the control group were injected with the same volume of corn oil on day 4 of pregnancy at 9 am. Seven days after RU486 injection, all mice were euthanized by inhalation of CO_2_ gas and both uterine horns were excised to determine the number of implantation sites. The number of implanted embryos on each uterine horn was recorded.

### Densitometry and statistical analysis

The intensity of the bands obtained from RT-PCR and western blot analysis, and the attached spheroids from the adhesion assay were quantified with ImageJ software. Data from all experiments are indicated as the percentage or fold compared to the control and expressed as the mean ± SD. The differences between the mean values of 2 groups were determined using a standard *t*-test with the GraphPad Prism (GraphPad Software, CA, USA). Furthermore, for comparison among groups, one-way analysis of variance (ANOVA) tests with a Dunnet’s post-hoc comparison were used with the assistance of GraphPad Prism. The minimum significance level was set at a *P* value of 0.05. All experiments were independently performed at least 3 times.

## Results

### Phytochemical validation of *P*. *lactiflora* extracts and effect on cell viability of Ishikawa cells

**Fig 1:**
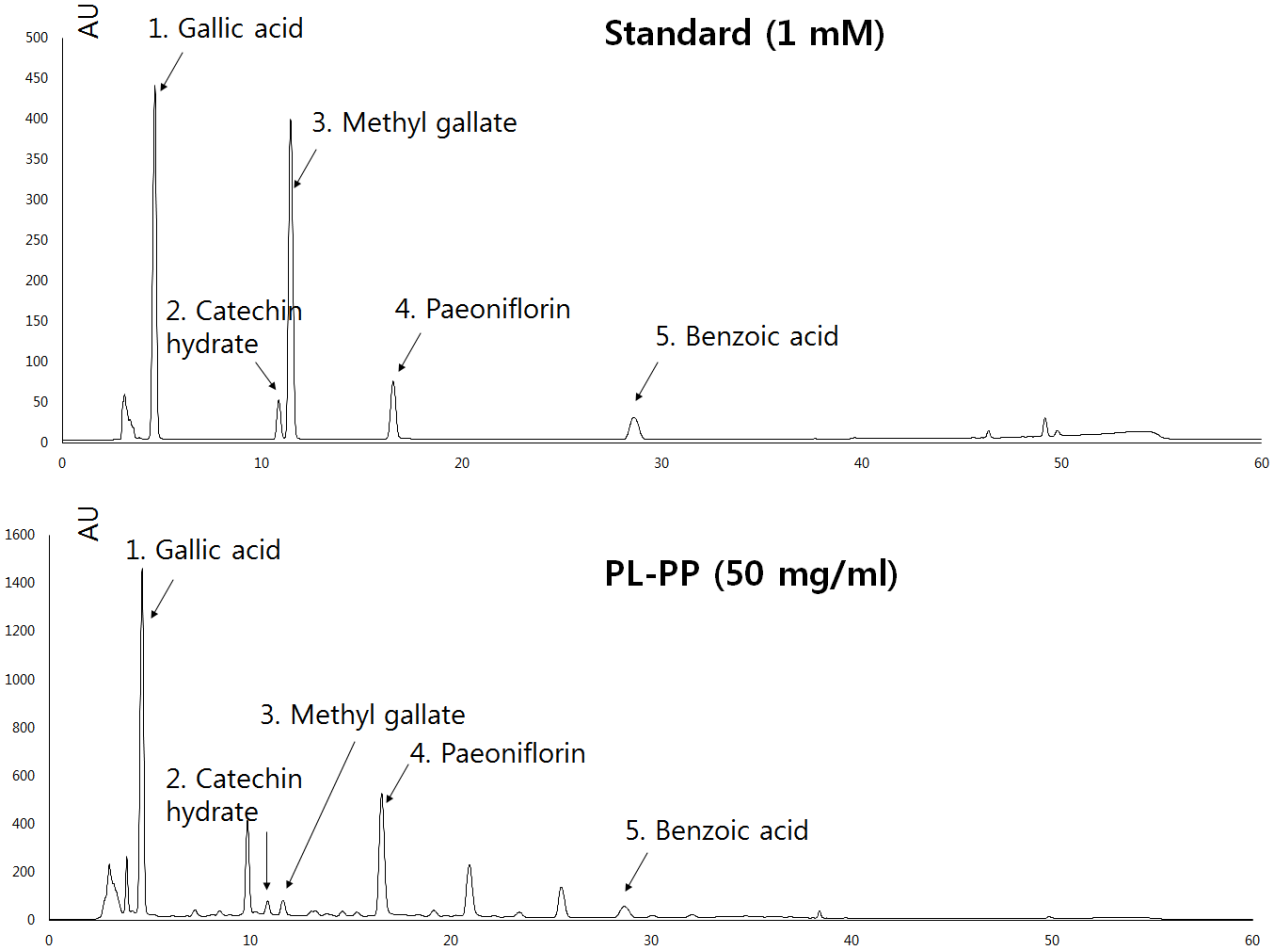
PL-PP HPL chromatogram. HPLC was performed as described in the Materials and Methods. Retention times of the standard compounds were 4.8, 10.5, 11.5, 17.5, and 28 min for gallic acid, catechin, methyl gallate, paeoniflorin, and benzoic acid, respectively.

As shown in Fig 1, PL-PP contained gallic acid, catechin, methyl gallate, paeoniflorin, and benzoic acid. PL-PP contains 151.14 μg/mg (>15%) paeoniflorin, which was the most abundant molecule among the five compounds tested (S1 Table).

As shown in S2A Fig, PL-WE treatment inhibited cell growth in a dose-dependent manner. Treatment with 10, 50, 100, and 500 μg/mL PL-WE showed 8, 22, 31, and 63% inhibition, respectively, while PL-PP treatment at concentrations of 500 μg/mL inhibited cell proliferation by 21% (S2B Fig). PL-PP did not have significant cytotoxic activity up to 100 μg/mL; therefore, we used PL-PP concentrations up to 50 μg/mL for the subsequent experiments.

### PL-PP increases LIF expression in Ishikawa cells and adhesion of JAr spheroids to Ishikawa cells

**Fig 2:**
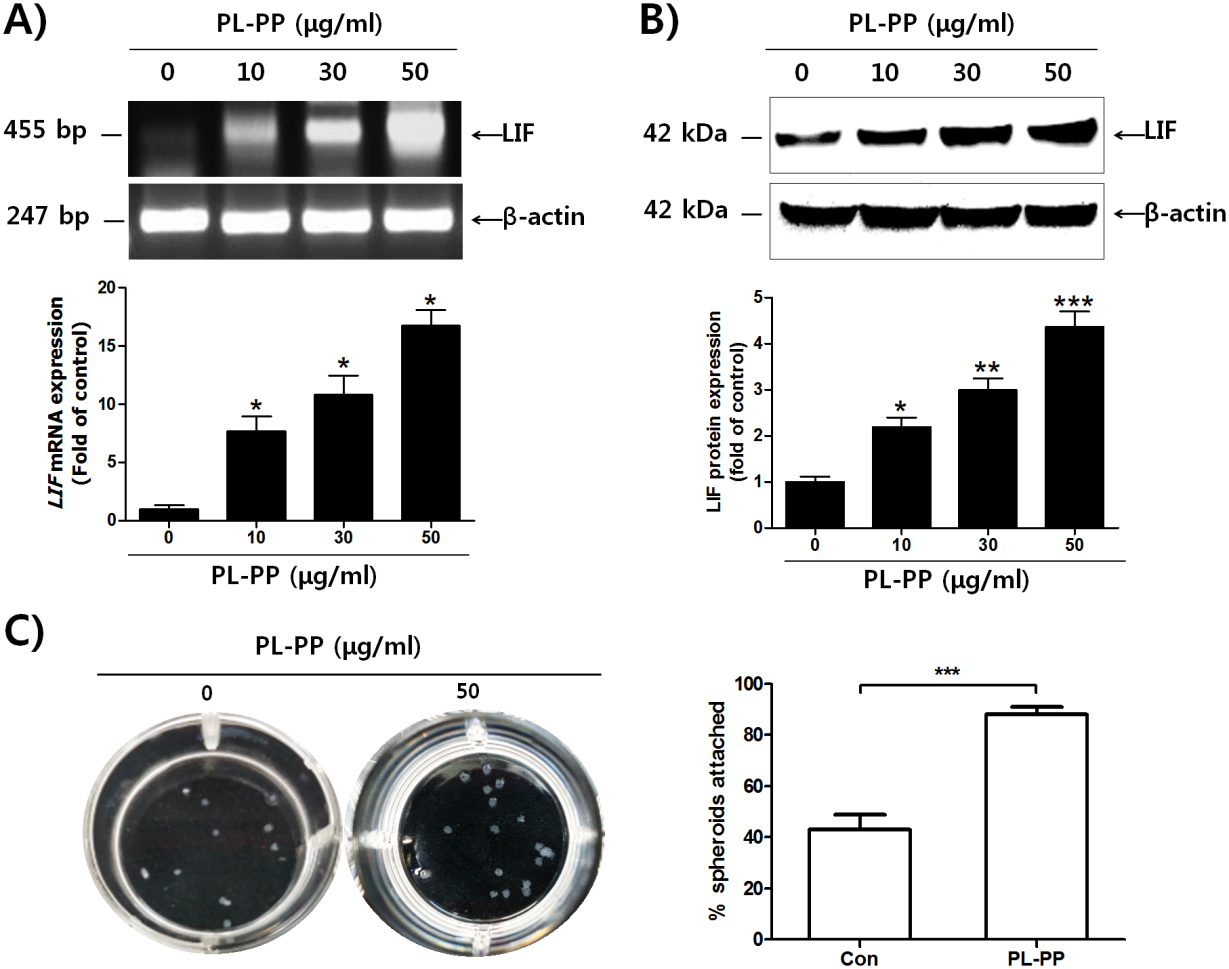
The effect of PL-PP on LIF expression and adhesion of JAr spheroids to Ishikawa cells. **(A, B)** Total RNA and protein were isolated after treatment with the indicated concentrations of PL-PP in serum—free medium for 24 h. Relative levels of LIF mRNA and protein were examined by RT-PCR and western blot analysis, respectively. The intensity of the band of interest was estimated by densitometric analysis and calculated as the mean ± SD of three independent experiments (* *P* < 0.05, ** *P* < 0.01, *** *P* < 0.001 compared to the control group). **(C)** Ishikawa cells were cultured in 24-well plates and treated with or without PL-PP (50 μg/mL) for 48 h. Twenty JAr spheroids were added onto the Ishikawa cell monolayer. The number of JAr spheroids bound to confluent Ishikawa cells was manually counted and calculated as the mean ± SD of three independent experiments (* *P* < 0.05 compared to each group).

Next, we investigated whether *P*. *lactiflora* extracts could induce LIF expression in Ishikawa cells. As shown in S3A and S3B Fig, *LIF* mRNA and protein levels were significantly increased by PL-PP treatment in Ishikawa cells. We also confirmed the increase in LIF protein expression in PL-PP-treated Ishikawa cells (S3B Fig). Ishikawa cells were treated with PL-PP at 10, 30, and 50 μg/mL doses. Both mRNA and protein levels increased in a dose-dependent manner (Fig 2A and 2B). We next examined whether PL-PP enhances the adhesion of Jar spheroids to Ishikawa cells. As shown in Fig 2C, the number of JAr spheroids adhered to PL-PP-treated Ishikawa cells (17.6/20, 88%) was significantly higher than that adhered to the control cells (8.6/20, 43%). PL-WE, on the other hand, did not affect the adhesion of JAr spheroids to Ishikawa cells (S4 Fig).

### PL-PP regulates LIF expression through the p38 and MEK/ERK signaling pathways

**Fig 3:**
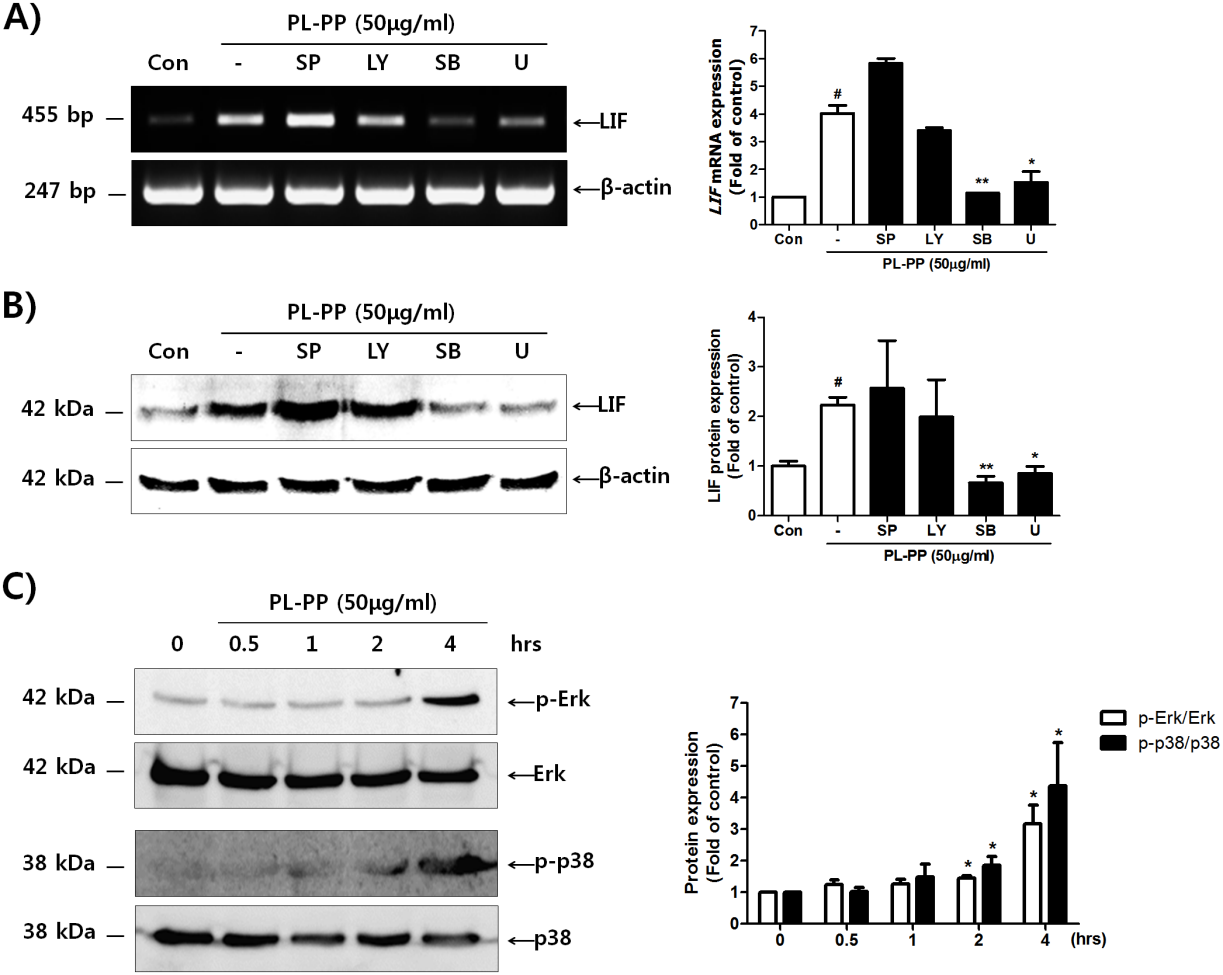
Identification of signaling pathways involved in PL-PP-induced LIF expression. Ishikawa cells were pre-treated with SP600125 (10 μM), LY294002 (15 μM), SB203580 (10 μM), and/or U0126 (0.5 μM) for 1 h. After treatment of each signaling inhibitor, the cells were stimulated with PL-PP (50 μg/mL) for 24 h. Total RNA and protein were isolated from each sample and LIF expression was evaluated by RT-PCR **(A)** and western blot analysis **(B)**. The intensity of the band of interest was estimated by densitometric analysis and calculated as means ± SD of two independent experiments (^**#**^ *P* < 0.01 vs. the untreated control. * *P* < 0.05 and ** *P* < 0.01 vs. the PL-PP-treated control). Ishikawa cells were treated with PL-PP for the indicated times (0, 0.5, 1, 2, and 4 h). Total protein was isolated from each sample and p38 and ERk phosphorylations were evaluated by western blot analysis **(C)**. The intensity of the band of interest was estimated by densitometric analysis and calculated as means ± SD of three independent experiments (* *P* < 0.05 compared to each control).

To determine the upstream signaling pathways responsible for PL-PP-induced LIF expression, we used various signaling inhibitors. The specific inhibitors of JNK (SP600125) and PI3K (LY294002) had no effect on the PL-PP-induced LIF expression (Fig 3A and 3B). However, p38MAPK (SB203580) and MEK/ERK inhibitors (U0126) did inhibit the PL-PP-induced LIF expression. As shown in Fig 3C, PL-PP markedly induced the phosphorylation of p38 and ERK. These results suggest that the activation of p38 and ERK, but not that of JNK or PI3K, is required for the PL-PP-induced expression of LIF.

### LIF knockdown inhibits adhesion of JAr spheroids to PL-PP-treated Ishikawa cells

**Fig 4:**
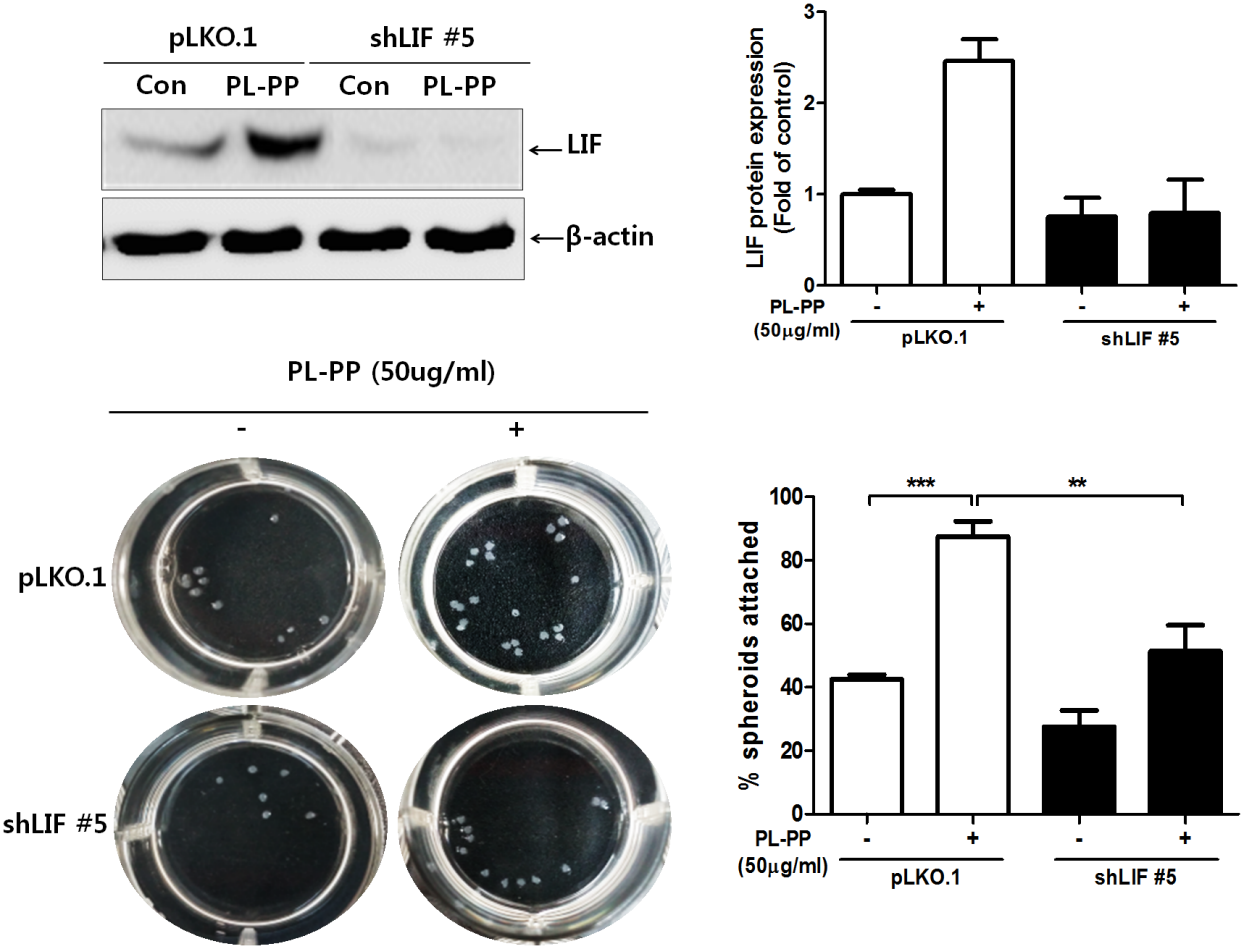
Adhesion of JAr spheroids to PL-PP-stimulated LIF-knockdown Ishikawa cells. Ishikawa cells harboring pLKO.1 or shLIF vector were cultured in 24-well plates and treated with or without PL-PP (50 μg/mL) in serum-free medium for 48 h. Twenty JAr spheroids were added onto the Ishikawa cell monolayer. Representative pictures were taken and the number of adhered JAr spheroids to Ishikawa cells was counted and calculated as the mean ± SD of three independent experiments (** *P* < 0.01 and *** *P* < 0.001 compared to each group).

To examine whether LIF plays an important role in promoting the adhesion of JAr spheroids to PL-PP-treated Ishikawa cells, we knocked down *LIF* expression in Ishikawa cells by shLIF lentiviral infection. The efficiency of knock-down was evaluated by LIF expression using RT-PCR (data not shown). Of STAT3, a down-stream effector of LIF signaling was found to be not activated by PL-PP treatment in LIF-knockdown cells (S5 Fig). As shown in Fig 4, the adhesion of JAr spheroids to PL-PP treated LIF-knockdown Ishikawa cells was significantly (10.25/20, 51, 25%) reduced compared to PL-PP-induced control Ishikawa cells (17.5/20, 87.5%). The data suggests that PL-PP-stimulated LIF gene expression may be necessary to facilitate the adhesion of Jar spheroids to Ishikawa cells.

### PL-PP positively regulates the expression of adhesion molecules such as integrin β3 and β5 in LIF-dependent manner

**Fig 5:**
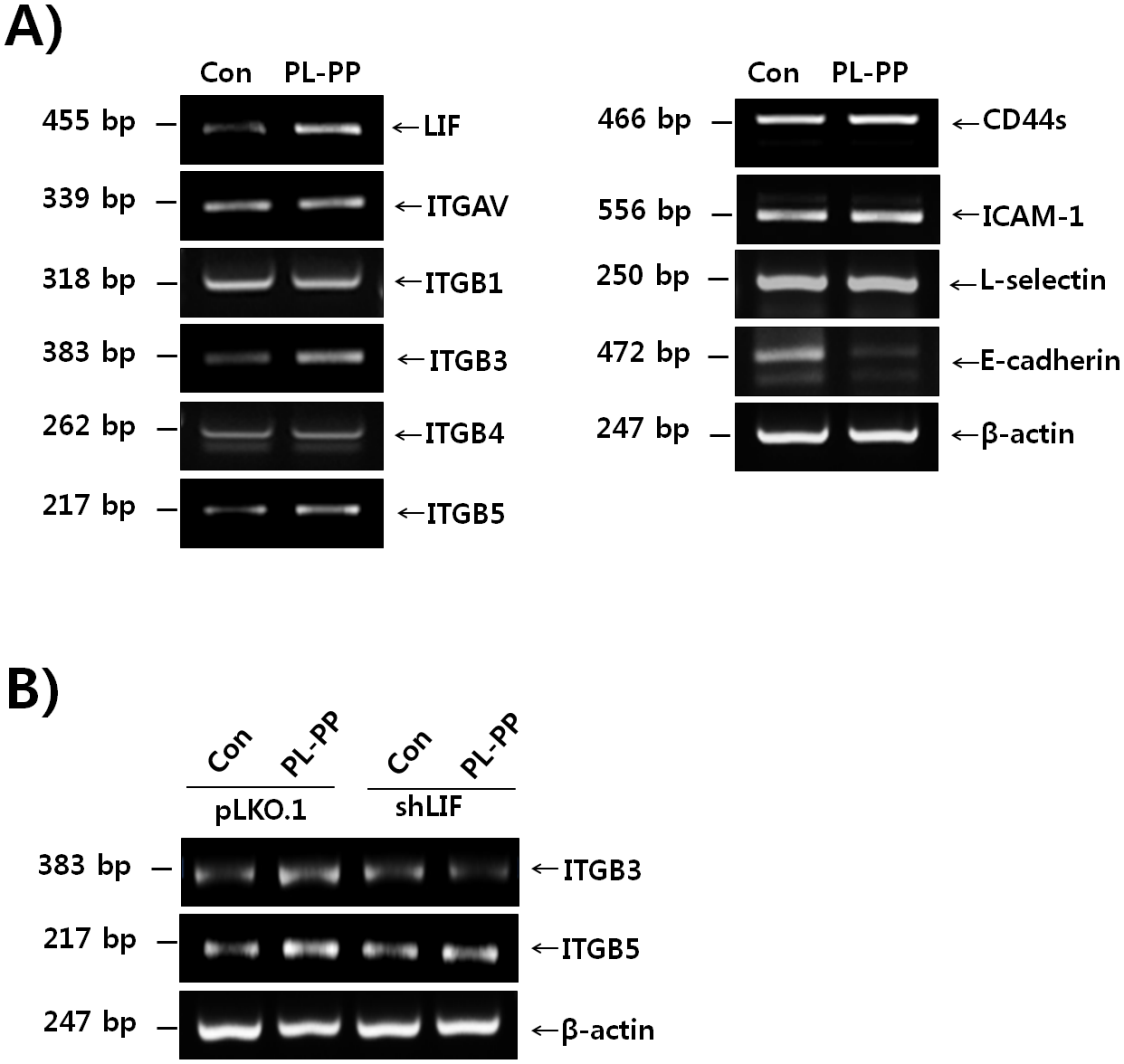
Expression of adhesion molecules in PL-PP treated Ishikawa cells. **(A)** Ishikawa cells were treated with or without PL-PP (50 μg/mL) for 24 h, and total RNA was extracted. The expression levels of *LIF*, *ITGAV*, *ITGB1*, *ITGB3*, *ITGB4*, *ITGB5*, *ICAM-1*, *L-selectin*, *E-cadherin*, and *CD44* mRNA were analysed by RT-PCR. β-actin was used as an internal control. **(B)** The pLKO.1 –or shLIF—transfected Ishikawa cells were treated with PL-PP (50 μg/mL) for 24 h. Total RNA was extracted and *ITGB3* and *ITGB5* mRNA expression levels were measured by RT-PCR. β-actin was used as an internal control.

We examined whether PL-PP has an effect on expression of various adhesion molecules using RT-PCR. As shown in Fig 5A, PL-PP induced the expression of integrin β3 (*ITGB3*) and *ITGB5*, but not of integrin αV (*ITGAV*), *ITGB1*, *ITGB4*, intercellular adhesion molecule 1 (*ICAM-1*), *L-selectin*, and *CD44* in Ishikawa cells. A decrease was observed in the mRNA levels of E-cadherin on treatment of Ishikawa cells with PL-PP (Fig 5A). PL-PP markedly increased the expression of *LIF* and *ITGB5* mRNA in non-receptive endometrial cell line- AN3-CA. However, the expression levels of other adhesion molecules such as integrin αV, β1, β3, β4, CD44, ICAM-1, L-selectin, and E-cadherin were not modulated by PL-PP treatment. PL-PP enhanced, thiugh not significantly, the adhesion of JAr spheroids to AN3-CA cells (S6 Fig).

To further investigate whether the PL-PP induced increase in the expression of adhesion molecules is mediated via a LIF-dependent pathway, we assessed *ITGB3* and *ITGB5* transcript levels in PL-PP treated pLKO.1- and shLIF-transfected Ishikawa cells. The transcript levels of *ITGB3* and *ITGB5* were found to be lower in PL-PP treated LIF knock-down cells compared with PL-PP treated pLKO.1-transfected cells (Fig 5B). These results suggest that PL-PP induces an increase in the expression of *ITGB3* and *ITGB5* through a LIF-dependent pathway.

### PL-PP administration restores implantation in an implantation-failure mouse model

**Fig 6:**
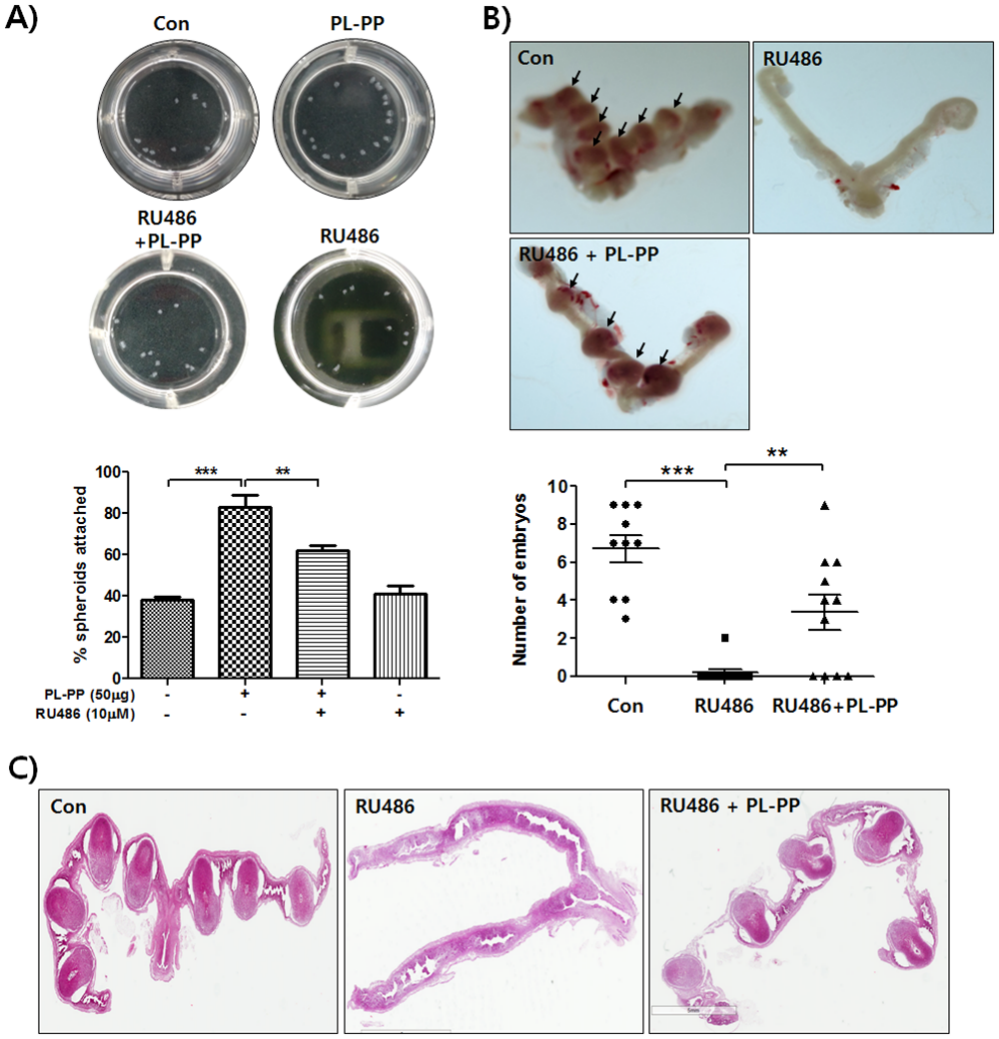
*In vitro* and *In vivo* effect of PL-PP on implantation in the presence of a progesterone antagonist. **(A)** Ishikawa cells in the absence or presence of RU486 (10 μM) were treated with or without PL-PP (50 μg/mL) in serum free medium for 48 h. Twenty JAr spheroids were added onto the Ishikawa cell monolayer. The number of adherent JAr spheroids to Ishikawa cells was counted in representative pictures and calculated as the mean ± SD of three independent experiments (* *P* < 0.05, *** *P* < 0.001 compared to each group and ns means no significance) **(B)** Female mice were treated with or without RU486 and PL-PP. After 7 days, all mice were euthanized and both uterine horns were excised to determine the number of implantation sites. Pictures of implanted embryos are shown. Arrows indicate implanted embryos. The number of implantation sites was counted and calculated as the mean ± SD of three independent experiments (** *P* < 0.05 and *** *P* < 0.001 compared to each group). **(C)** The uterin tissue sections were analyzed histologically after H&E staining. Embryo implantation sites are indicated by arrows.

**Fig 7:**
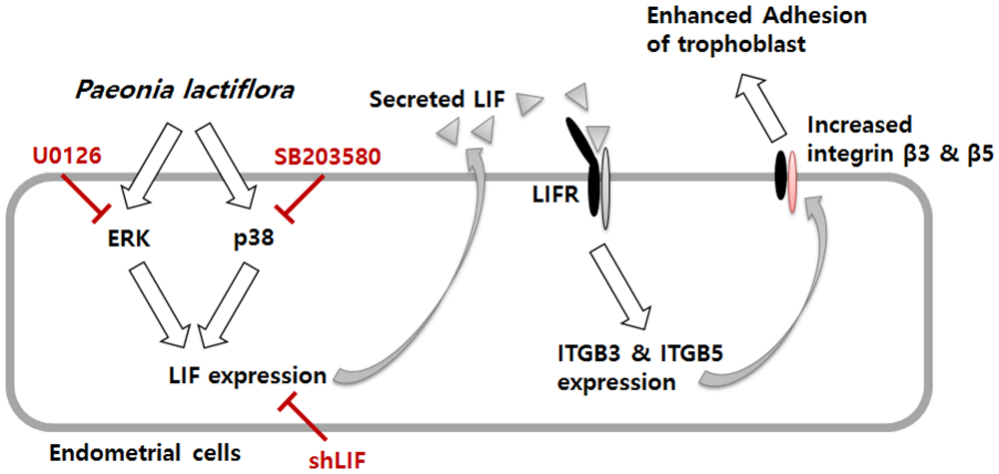
Schematic representation of the mechanism underlying the *P*. *lactiflora* induced LIF expression in the endometrium and its relevance. *P*. *lactiflora* induces LIF expression through the activation of p38 and MEK/ERK pathways, leading to an increase in the trophoblast adhesion to the endometrium. Inhibition of LIF expression blocks the expression of adhesion molecules, such as integrin β3 and β5, and adhesion of the trophoblast to the endometrium.

We next investigated whether the administration of PL-PP could effectively promotes blastocyst implantation *in vivo*. First, we assessed the effect of RU486, a well-known contraceptive drug, on LIF expression in human endometrial Ishikawa cells. RU486 treatment did not have any significant effect on LIF expression (data not shown). We also examined the *in vitro* effect of PL-PP on the adhesion of JAr spheroids to RU486 treated-Ishikawa cells. As shown in Fig 6A, the number of JAr spheroids adhered to RU486-pretreated Ishikawa cells increased from 41% to 61.66% after treatment with PL-PP. Further, the total number of implanted embryos in the RU486 with PL-PP group (3.36 ± 3.07), though lower than that in the control group (6.70 ± 2.26), was significantly higher than that in the RU486 group (0.18 ± 0.60). This indicated that PL-PP restores the implantation defect induced by RU486 treatment. Taken together, the data suggest that PL-PP-induced *LIF* expression plays a crucial role in the adhesion of Jar spheroids to the endometrium (Fig 7).

## Discussion

Embryo quality and endometrial receptivity are crucial factors for successful implantation. In this study, we investigated the potential of medicinal herbs to improve uterine receptivity. Although *P*. *lactiflora* is one of the most well-known herbs for the treatment of diverse gynecological diseases [21,22], the effects of *P*. *lactiflora* on endometrial receptivity have not been investigated. PL-WE, which is extracted from the roots of *P*. *lactiflora* with boiling water, contains polysaccharides. It has been reported that polysaccharides cause an abnormal cellular immune response and interruption of cell-cell interactions [29,30]. In the present study, PL-WE did not induce LIF expression in the endometrium and was found to be cytotoxic. PL-PP was devoid of polysaccharides. Biochemical analysis suggests that paeoniflorin is one of the major compounds contributing to the effects of *P*. *lactiflora*.

Several factors such as integrins, interleukin-1 (IL-1), calcitonin, amphiregulin, epidermal growth factor (EGF), HB (heparin binding)-EGF, colony stimulating factor-1, LIF, mucins, trophinin/tastin, Hoxa-10, and cyclooxygenase (COX) contribute to endometrial receptivity [31]. LIF, a key factor for embryo implantation, is expressed in the glandular epithelial, luminal epithelial and stromal cells of the endometrium [5,32]. Our investigations demonstrated that i*n vitro* PL-PP treatment increases the LIF expression and the adhesion of Jar spheroids to Ishikawa cells. Further *LIF* knockdown inhibited the adhesion of JAr spheroids to PL-PP-treated Ishikawa cells. These results suggest that an increase in the LIF expression in response to PL-PP favors the adhesion of JAr spheroids to Ishikawa cells.

Interleukin-1 (IL-1) and tumor necrosis factor-α (TNF-α) induce the phosphorylation of nuclear factor κB (NF-κB), which binds to the corresponding responsive elements in the *LIF* promoter region [33]. IL-1β stimulated LIF induction is mediated by MEK/ERK and NF-κB-pathways in normal articular human chondrocytes [34]. TNF-α is known to induce LIF expression through p38MAPK signaling pathway in bone marrow stromal cells from pediatric patients with myelodysplastic syndrome and neuronal retinal cells [35,36]. Our results showed involvement of p38 and MEK/ERK signaling pathways in PL-PP-induced LIF expression. It has been reported that the inhibition of LIF-induced STAT3 activation in the endometrium suppresses implantation [37]. We also observed that the PL-PP induced increase in LIF expression involves STAT3 activation.

Implantation is associated with modulation in the endometrial expression of several adhesion molecules, including integrins [38–40]. Integrins α2β1, α3β1, and α6β4 are reported to be constitutively expressed in Ishikawa cells. Further the expression of α1β1 and αvβ3 integrins is reported to be under hormonal regulation [41]. The cell surface expression of adhesion molecules determines the adhesiveness of JAr spheroids to endometrial cells [42]. Human endometrial RL95-2 cells, known as highly adhesive cells, express integrin α6, β1, and β4 subunits. On the other hand, non-receptive human endometrial AN3-CA cells lack β4 integrin subunit and show reduced expression of α6 and β1 integrin subunits. This results in lesser adhesion of JAr spheroids to endometrial AN3-CA cells [43].

E-cadherin is located on the plasma membrane of the epithelial cells and is critical for the maintenance of adherent junctions [44–46]. It has been reported that E-cadherin expression is reduced in the uterine epithelium during implantation in rodents [47]. This results in the reorganization of the adherent junctions between epithelial cells to facilitate blastocyst implantation [47]. Our data also demonstrated a reduction in E-cadherin expression in endometrial cells, following PL-PP treatment. Further the study demonstrated that PL-PP induces the expression of integrins β3 and β5 in a LIF-dependent manner.

Danielsson *et al*. showed that RU486 treatment immediately post ovulation reduces endometrial glandular LIF expression at the expected time of implantation [48]. Lalitkumar *et al*. reported that RU486 inhibits human blastocyst attachment to an endometrial three-dimensional cell model *in vitro* [49]. A previous report showed that various cytokines such as EGF, TGF-α, PDGF, and IL-1β are potent inducers of LIF expression in endometrial stromal cells [48]. However, steroid hormones such as estradiol and progesterone do not exert a regulatory effect on LIF expression in human endometrial cells [50]. We also did not observe any adverse effect of RU486 on LIF expression in Ishikawa cells. Also the number of JAr spheroids attached to Ishikawa cells did not change due to pre-treatment with RU486. PL-PP treatment also augmented the number of implanted embryos in an *in vivo* implantation failure model, developed using RU486. It is likely that a PL-PP induced increase in LIF expression does not involve the progesterone receptor signaling pathway.

## Conclusions

PL-PP increases the LIF expression in Ishikawa cells through p38 MAPK and MEK/ERK signaling pathways in endometrial Ishikawa cells. The increase in LIF expression by PL-PP favors the adhesion of human trophoblastic JAr spheroids to Ishikawa cells. Also, the administration of PL-PP improves embryo implantation in RU486-treated mice. These results suggest that PL-PP may facilitate the adhesion of the blastocyst to the endometrium. PL-PP may be considered as a potential therapeutic herbal medicine for improving pregnancy rates.

## Supporting Information

S1 Fig Schematic diagram of the extraction procedure from the roots of *P*. *lactiflora*. (TIF)

S2 Fig Cytotoxic effect of PL-WE and PL-PP on Ishikawa cells. Twenty-thousand cells were cultured in 12-well plates with the indicated concentrations of **(A)** PL-WE and **(B)** PL-PP. Cell viability was estimated 24 h after treatment using an MTT assay. Data represent the mean ± SD of three independent measurements (* *P* < 0.05 and *** *P* < 0.001 compared to the control group).
(TIF)

S3 Fig Effects of PL-WE or PL-PP on LIF expression in Ishikawa cells. **(A, B)** Ishikawa cells were treated with PL-WE or PL-PP (50μg/mL) in serum-free medium for 24 h. Total RNA and protein were extracted from PL-WE- or PL-PP-treated Ishikawa cells. LIF expression was measured by RT-PCR and western blot analysis. The intensity of the band of interest was estimated by densitometric analysis, and calculated as the mean ± SD of three independent experiments (** *P* < 0.01 compared to each group).
(TIF)

S4 Fig Effect of PL-WE on the adhesion of JAr spheroids to Ishikawa cells. Ishikawa cells were cultured in 6-well plates and treated with or without PL-WE (50 μg/mL) for 48h. Twenty JAr spheroids were added onto the Ishikawa cell monolayer. The number of JAr spheroids bound to confluent Ishikawa cells was manually counted, and calculated as the means ± SD of three independent experiments (* *P* < 0.05 compared to each group).
(TIF)

S5 Fig Effect of PL-PP on the activation of STAT3 in Ishikawa cells. Ishikawa cells transfected with pLKO.1 and shLIF were treated with or without PL-PP (50 μg/mL) in serum-free medium for 24 h. The levels of STAT3 and p-STAT3 proteins were measured by Western blot analysis. The intensity of the band of interest was estimated by densitometric analysis and calculated as the mean ± SD of three independent experiments (* *P* < 0.05 compared to each group).
(TIF)

S6 Fig The expression levels of the adhesion molecules by PL-PP in non-receptive endometrial AN3-CA cells and the adhesion of JAr spheroids to PL-PP -treated AN3-CA cells. **(A)** AN3-CA cells were treated with or without PL-PP (50 μg/mL) for 24 h, and total RNA was extracted. The expression levels of *LIF*, *ITGAV*, *ITGB1*, *ITGB3*, *ITGB4*, *ITGB5*, *ICAM-1*, *L-selectin*, *E-cadherin* and *CD44* mRNA were examined by RT-PCR. β-actin was used as an internal control. **(B)** AN3-CA cells were cultured in 24-well plates and treated with or without PL-PP (50 μg/mL) in serum-free medium for 48 h. Twenty JAr spheroids were added onto the Ishikawa cell monolayer. The number of adherent JAr spheroids to Ishikawa cells was counted in representative pictures, and calculated as the mean ± SD of three independent experiments (** *P* < 0.01 compared to each group).
(TIF)

S1 Table Quantitative phytochemical constituents of PL-PP. (DOCX)
