# Supplementary figures and images for: Paeonia lactiflora Enhances the Adhesion of Trophoblast to the Endometrium via Induction of Leukemia Inhibitory Factor Expression
Source: PLoS One. 2016 Feb 3;11(2):e0148232. doi: 10.1371/journal.pone.0148232 (PMC4739624; doi:10.1371/journal.pone.0148232)

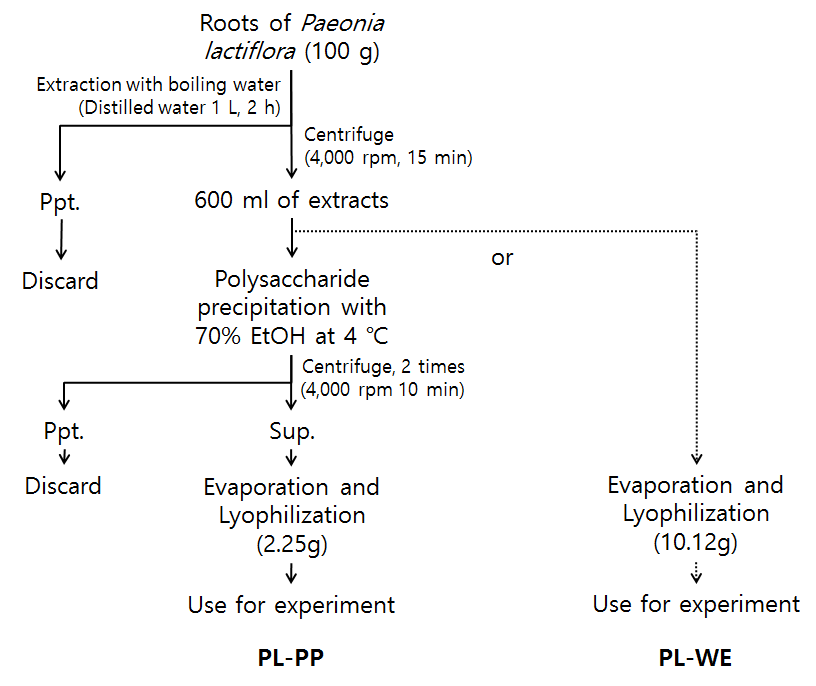

Supplement: S1 Fig — (TIF) [file pone.0148232.s001.tif]

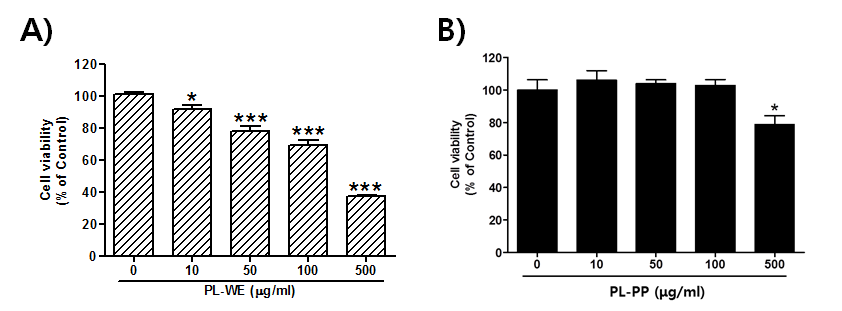

Supplement: S2 Fig — Twenty-thousand cells were cultured in 12-well plates with the indicated concentrations of (A) PL-WE and (B) PL-PP. Cell viability was estimated 24 h after treatment using an MTT assay. Data represent the mean ± SD of three independent measurements (* P < 0.05 and *** P < 0.001 compared to the control group). (TIF) [file pone.0148232.s002.tif]

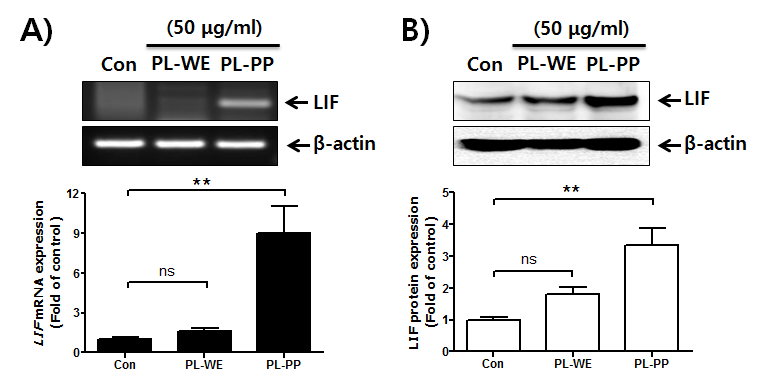

Supplement: S3 Fig — (A, B) Ishikawa cells were treated with PL-WE or PL-PP (50μg/mL) in serum-free medium for 24 h. Total RNA and protein were extracted from PL-WE- or PL-PP-treated Ishikawa cells. LIF expression was measured by RT-PCR and western blot analysis. The intensity of the band of interest was estimated by densitometric analysis, and calculated as the mean ± SD of three independent experiments (** P < 0.01 compared to each group). (TIF) [file pone.0148232.s003.tif]

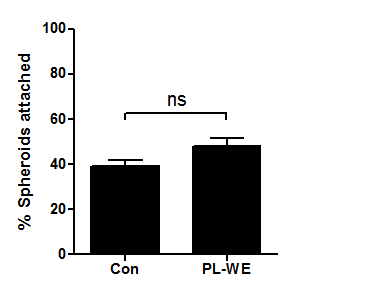

Supplement: S4 Fig — Ishikawa cells were cultured in 6-well plates and treated with or without PL-WE (50 μg/mL) for 48h. Twenty JAr spheroids were added onto the Ishikawa cell monolayer. The number of JAr spheroids bound to confluent Ishikawa cells was manually counted, and calculated as the means ± SD of three independent experiments (* P < 0.05 compared to each group). (TIF) [file pone.0148232.s004.tif]

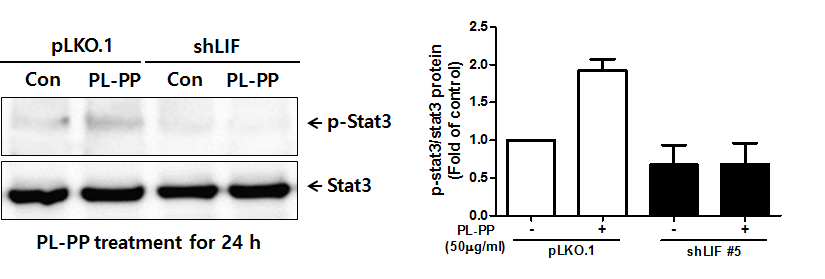

Supplement: S5 Fig — Ishikawa cells transfected with pLKO.1 and shLIF were treated with or without PL-PP (50 μg/mL) in serum-free medium for 24 h. The levels of STAT3 and p-STAT3 proteins were measured by Western blot analysis. The intensity of the band of interest was estimated by densitometric analysis and calculated as the mean ± SD of three independent experiments (* P < 0.05 compared to each group). (TIF) [file pone.0148232.s005.tif]

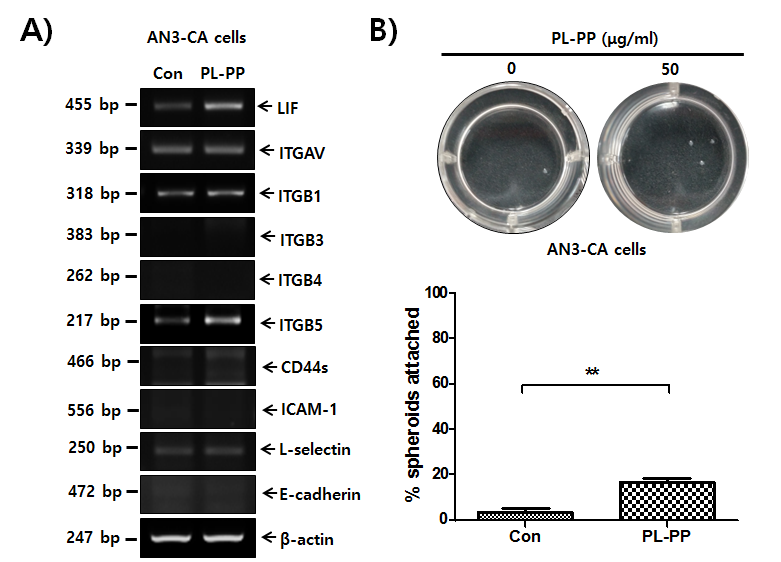

Supplement: S6 Fig — (A) AN3-CA cells were treated with or without PL-PP (50 μg/mL) for 24 h, and total RNA was extracted. The expression levels of LIF, ITGAV, ITGB1, ITGB3, ITGB4, ITGB5, ICAM-1, L-selectin, E-cadherin and CD44 mRNA were examined by RT-PCR. β-actin was used as an internal control. (B) AN3-CA cells were cultured in 24-well plates and treated with or without PL-PP (50 μg/mL) in serum-free medium for 48 h. Twenty JAr spheroids were added onto the Ishikawa cell monolayer. The number of adherent JAr spheroids to Ishikawa cells was counted in representative pictures, and calculated as the mean ± SD of three independent experiments (** P < 0.01 compared to each group). (TIF) [file pone.0148232.s006.tif]
