# Supplementary material for: Paeonia lactiflora Enhances the Adhesion of Trophoblast to the Endometrium via Induction of Leukemia Inhibitory Factor Expression
Source: PLoS One. 2016 Feb 3;11(2):e0148232. doi: 10.1371/journal.pone.0148232 (PMC4739624; doi:10.1371/journal.pone.0148232)
Supplement: S1 Table — (DOCX) [file pone.0148232.s007.docx]

S1 Table. Quantitative phytochemical constituents of PL-PP

| Phytochemicals | µg/mg extract |
| --- | --- |
| Benzoic acid | 3.86 |
| Catechin | 3.69 |
| Gallic acid | 20.90 |
| Methyl gallate | 7.75 |
| Paeoniflorin | 151.14 |
